# Security Architecture for Defining and Enforcing Security Profiles in DLT/SDN-Based IoT Systems

**DOI:** 10.3390/s20071882

**Published:** 2020-03-28

**Authors:** Sara N. Matheu, Alberto Robles Enciso, Alejandro Molina Zarca, Dan Garcia-Carrillo, José Luis Hernández-Ramos, Jorge Bernal Bernabe, Antonio F. Skarmeta

**Affiliations:** 1 Department of Information and Communications Engineering, University of Murcia, 30100 Murcia, Spain; alberto.roblese@um.es (A.R.E.); alejandro.mzarca@um.es (A.M.Z.); jorgebernal@um.es (J.B.B.); skarmeta@um.es (A.F.S.); 2 Odin Solutions, Department of Research and Innovation, Alcantarilla, 30820 Murcia, Spain; dgarcia@odins.es; 3 European Commission, Joint Research Centre, 21027 Ispra, Italy; jose-luis.hernandez-ramos@ec.europa.eu

**Keywords:** internet of things, security, SDN, MUD, blockchain, security policies

## Abstract

Despite the advantages that the Internet of Things (IoT) will bring to our daily life, the increasing interconnectivity, as well as the amount and sensitivity of data, make IoT devices an attractive target for attackers. To address this issue, the recent Manufacturer Usage Description (MUD) standard has been proposed to describe network access control policies in the manufacturing phase to protect the device during its operation by restricting its communications. In this paper, we define an architecture and process to obtain and enforce the MUD restrictions during the bootstrapping of a device. Furthermore, we extend the MUD model with a flexible policy language to express additional aspects, such as data privacy, channel protection, and resource authorization. For the enforcement of such enriched behavioral profiles, we make use of Software Defined Networking (SDN) techniques, as well as an attribute-based access control approach by using authorization credentials and encryption techniques. These techniques are used to protect devices’ data, which are shared through a blockchain platform. The resulting approach was implemented and evaluated in a real scenario, and is intended to reduce the attack surface of IoT deployments by restricting devices’ communication before they join a certain network.

## 1. Introduction

The exponential growth of the Internet of Things (IoT) brings endless possibilities to improve our daily lives based on the data exchanged among interconnected devices and systems. Indeed, the increasing digitalization of our surrounding environment is intended to improve existing deployments in different sectors, such as transport systems, agriculture, and energy management. However, the cost and time manufacturing restrictions, as well as the inherent constraints of these types of devices, such as computation power or memory, could lead to poor or even non-existent security mechanisms. Indeed, this situation has been exploited to launch different attacks, such as the well-known IoT botnets [1], to jointly attack specific targets.

One of the main initiatives to enhance security aspects in IoT devices is the usage of behavioral profiles, which describe the intended behavior of a device. Indeed, the realization of this concept has been mainly driven by the recent Manufacturer Usage Description (MUD) [2], which is an IETF standard encouraging manufacturers to represent the allowed/denied network communications of their devices through a simple and flexible data format. The behavioral profiles can be used to configure the device before it joins the network, in order to reduce the attack surface as well as to monitor its behavior to detect suspicious behaviors (e.g., due to an ongoing attack). MUD standard has attracted the attention of different Standards Developing Organization (SDOs), such as the National Institute Standards and Technology (NIST) in U.S. [3,4] that has proposed the creation of a vulnerability behavior database based on this standard (https://www.nist.gov/itl/applied-cybersecurity/nist-initiatives-iot). One of the strong points of the MUD standard is the potential integration with the Software-Defined Networking (SDN) paradigm for the automated and dynamic enforcement of the restrictions included in a MUD profile as discussed by Hamza et al. [5] and Ranganathan [6] by using OpenFlow [7].

Based on this, this work proposes an architecture and process to obtain and enforce the policies described in an extended MUD profile. In particular, we extend the MUD model to be able to specify a wider range of security policies. Although in a previous paper we proposed a MUD extension [8], it was limited to access control policies and specific security aspects. The extension proposed in this paper integrates the MUD model with the usage of the Medium-level Security Policy Language (MSPL), which has been used in the scope of the EU H2020 project ANASTACIA [9]. It provides a high expressiveness to specify security policies beyond network layer at medium-level abstraction (e.g., with information of the endpoints and communications, such as IP addresses and protocols), in an agnostic way to the enforcement process. Based on this, we extend our architecture proposed in [10], in which the MUD management was integrated in the bootstrapping process of the device (i.e., when the device joins a certain network). This way, MUD restrictions are enforced before the device is connected to the network, thus reducing the attack surface. For this, we integrate the MUD management and enforcement process with a lightweight bootstrapping approach based on the Constrained Application Protocol (CoAP) as a lower layer protocol of the Extensible Authentication Protocol (EAP) [11]. The resulting architecture is intended to enforce the extended MUD profiles based on the model extension. In particular, we show the definition and enforcement of network access control policies through SDN technologies, including restrictions about the communication itself. Furthermore, we define data privacy policies to protect the access to sensitive data, which are enforced through Ciphertext-Policy Attribute-Based Encryption (CP-ABE) [12], as well as restrictions over devices’ resources through an extension of CBOR Web Tokens (CWT) [13] and the eXtensible Access Control Markup Language (XACML) [14]. In addition, we propose the usage of blockchain to share devices’ information to different authorized devices and users.

In summary, the contributions of this paper are manifold:

- Extension of the current MUD model with MSPL to define a wide variety of security policies beyond network access control
- Integration of the MUD obtaining and enforcement processes in a lightweight bootstrapping process based on CoAP-EAP [15]
- Definition of the architecture and message exchange required to obtain and enforce MUD restrictions, improving security aspects of IoT devices’ operation
- Integration of SDN techniques with attribute-based security for the enforcement of the extended MUD restrictions
- Usage of the Distributed Ledger Technology (DLT) (e.g., blockchain) technology to ensure accountability and data provenance features for IoT devices and InterPlanetary File System (IPFS) technology to improve the scalability of the blockchain.
- Implementation and validation of the proposal in a real testbed showing its feasibility and performance to manage security profiles in IoT deployments

The remainder of this paper is organized as follows. Section 2 provides a review of the current literature related with the definition and enforcement of behavioral profiles in the context of IoT. Section 4 details the MUD standard as well as the extension proposed to represent additional types of security policies. Section 5 defines the proposed architecture, whereas Section 6 details the different processes and steps to manage the extended MUD profiles. Then, Section 7 describes the performance evaluation of the different processes. Finally, Section 8 concludes the paper and provides an outlook about our future work in this area.

## 2. Related Work

Behavioral profiles can help to reduce the attack surface and mitigate security attacks by enforcing security policies as well as monitoring the device’s expected behavior [16]. Traditionally, policy-based approaches have been used to specify the allowed/denied communications from/to a certain system. One of the most significant approaches is the Policy Core Information Model (PCIM) [17] standard from the IETF, which was used in several European projects focused on policy frameworks, such as PoSecCo (https://cordis.europa.eu/project/id/257129) and POSITIF (https://cordis.europa.eu/project/id/002314), to represent firewall and channel protection configurations, due to its high expressiveness. Other approaches have been proposed in last years, such as those proposed by Molloy et al. [18] and by Barrera et al. [19], which define a novel policy enforcement architecture to restrict the network behavior of IoT devices. Another more recent standard to specify these policies is the YANG Data Model for Network Access Control Lists (ACLs) [20], which is considered in the MUD standard [2]. MUD gives the responsibility to the manufacturer to specify the expected network behavior of an IoT device, describing the communications allowed to/from the device. It uses the mentioned YANG standard to represent the network behavior by using JSON [21] for serialization.

The MUD standard has attracted the attention of different SDOs. Recent NIST reports support the use of MUD [3,4], and consider the creation of a National Thing Behavior Database (NTBD) (https://www.nist.gov/itl/applied-cybersecurity/nist-initiatives-iot) based on it. One of the main advantages of MUD is the potential combination with SDN technologies in order to enforce the network restrictions described in a MUD file. This integration has been already considered in current literature. In particular, Hamza et al. [22] proposed an SDN-based architecture to translate MUD policies into flow rules to be enforced. These rules are used to detect attacks by using an Intrusion Detection System (IDS). The same authors [5] also proposed an SDN-based approach to monitor the MUD behavior of a device. An SDN-based framework was also considered by Al Shaboti et al. [23] to enforce network access control policies and mitigate spoofing attacks in the scope of smart homes. Furthermore, the MUD standard allows future potential extensions, for example, to consider Quality of Service (QoS) aspects of the communications.

Although the MUD model provides a standardized and flexible way to specify network policies, the provided semantics does not allow the specification of more fine-grained security aspects and other types of security policies beyond network access control. In this work, we extend the MUD model integrating the MSPL language to describe additional security aspects, which could be relevant to protect an IoT device, as well as detect and mitigate potential attacks. In particular, we describe the extension and enforcement of four different types of policies: extended network access control to restrict the communications of the device; channel protection, intended to apply fine-grained restrictions on a device’s communications; authorization, which is intended to protect the access to devices’ resources; and data privacy, which is used to encrypt the data provided by a device. In this context, while SDN technologies have been considered in the literature to enforce MUD restrictions, the enforcement of such additional restrictions requires the integration of additional mechanisms.

Regarding authorization, the eXtensible Access Control Markup Language (XACML) [14] is the de facto standard to define and enforce authorization restrictions. Indeed, we used XACML in our previous works (e.g., [24,25]) to foster a high degree of interoperability among different vendors’ access control implementations. A complementary and well-known approach to enforce authorization restrictions is based on the usage of authorization tokens (e.g., OAuth [26]). In the context of IoT, OAuth is being considered in the scope of the IETF Authentication and Authorization for Constrained Environments (ACE) Working Group (https://datatracker.ietf.org/wg/ace/documents/) by using the Concise Binary Object Representation (CBOR) standard [27] to represent CBOR Web Tokens (CWT) [13]. This way, the token size is reduced, making it suitable for constrained devices. In this paper, we combine the usage of CWT with AIF [28] to embed the access rights over resources in the token. Furthermore, we use XACML as a policy-based infrastructure to generate (or not) a certain CWT-AIF token based on the evaluation of authorization policies.

Focused on data privacy aspects, it is important to ensure that only legitimate and authorized users are able to access the data provided by IoT devices, in order to avoid any potential privacy leakage. In this direction, Sarkar et al. [29] proposed a layered IoT architecture for security and privacy that uses access control policy models, based on the Seckit tool [30]. Seckit allows representing different security requirements of the IoT behavior by using high level meta-models. The approach is based on the Message Queuing Telemetry Transport (MQTT) protocol [31] to enforce such requirements. Furthermore, the use of encryption techniques could mitigate the emergence of privacy issues by protecting the data generated and shared by IoT devices. A flexible cryptographic approach is represented by the Ciphertext-Policy Attribute-Based Encryption (CP-ABE) scheme, which uses an attribute-based policy to encrypt data, so that only devices or users satisfying this policy will decrypt such data. In this paper, we enforce data privacy aspects by using CP-ABE. In particular, we use the attributes defined in our extended MUD model to encrypt data.

Current research also addresses other behavioral aspects of IoT devices beyond the mentioned security policies. The proposed framework *P4SINC* by Phung et al. [32] represents a general execution policy framework to enforce restrictions during the operation phase of IoT devices. Ontology-based languages such as KAoS [33] or rei [34] have been also considered in current literature. However, whereas some of these languages are focused on a specific type of security policy, others are excessively complex to be employed by non-expert users. Dealing with this, Valenza et al. [35] described two different general policy languages, which were defined in the context of the EU SECURED project (https://www.secured-fp7.eu/). On the one hand, the High-level Security Policy Language (HSPL) is focused on the description of security requirements for non-expert users. On the other hand, the Medium-level Security Policy Language (MSPL) is oriented to more technical users, providing a low abstraction to represent security configurations in a generic way.

Based on this, our proposal extends the MUD model integrating the MSPL language to describe additional security aspects (e.g., authorization and data privacy), beyond network access control. To manage the extended MUD profiles, we propose a security architecture that integrates the management of MUD profiles (including the steps for obtaining and enforcing the restrictions included in the MUD file) in the device’s bootstrapping. Towards this end, we use an integrated approach of access control approaches and SDN techniques. Furthermore, we leverage existing approaches that use CP-ABE in blockchain [36], adapting them for IoT constrained deployments in which devices can delegate CP-ABE operations to an intermediate proxy. Blockchain offers accountability and data provenance features for our IoT solution [37], which, when combined with CP-ABE, can help to cope with blockchain privacy-preservation challenges [38]. In this regard, our architecture provides a policy-based, privacy-preserving, accountable, and fine-grained data sharing solution for IoT constrained deployments.

## 3. Motivation, Challenges, and Proposed Solutions

As discussed in Section 1, the continuous attacks leveraging the weaknesses associated with IoT devices require measures to *prevent and reduce the attack surface*, protecting both these devices and the networks in which they interact. In this sense, the definition of behavioral profiles could be considered as a promising mechanism to define the expected behavior of devices in order to enforce security restrictions over them (e.g., reducing the communication endpoints), as well as to detect suspicious behaviors that could lead to an attack. Toward this end, *standardized and widely accepted mechanisms* are crucial to homogenize and harmonize the definition and management of such profiles, in order to foster secure and interoperable deployments. For this reason, the MUD standard could be considered as a relevant effort to define the devices’ intended behavior during the manufacturing phase. The aim of the MUD standard [2] is to reduce the attack surface of a certain specific-purpose device by delegating the task of creating a behavioral profile to the manufacturers rather than a network administrator. Some examples of these restrictions could be “allow communications between devices of the same manufacturer” or “deny the access to a specific service though a specific port”. However, as discussed in Section 2, while the MUD data model provides a standardized and flexible way to specify network policies, the provided semantics does not allow the specification of more fine-grained security aspects, leading to a *lack of expressiveness beyond network restrictions*. In this paper, we extend the MUD model with the aim to represent different kinds of security policies beyond network access control restrictions. In particular, the extended MUD is able to describe four types of security policies:

- Extended network access control (filtering) policies restrict the communications from/to the device at network layer. Although the standard MUD is aimed to describe these policies, the proposed extension adds more fine-grained conditions (e.g., MAC address or interface).
- Channel protection aims to specify fine-grained security aspects of devices’ communications such as the ciphersuite to be used by a certain protocol (e.g., the Datagram Transport Layer Security (DTLS) protocol [39]).
- Data privacy is intended to specify combination of attributes to encrypt the data provided by IoT devices. Therefore, those entities that possess the necessary attributes specified in the extended MUD profile, can access to the encrypted data.
- Authorization over resources is focused on protecting the access to devices’ resources. These policies describe the authorized actions over a resource, as well as the entity that is allowed to do that.

As a result of the proposed extension, a new challenge arises related with *the enforcement of the extended security policies*. Indeed, the MUD standard does not define the required components and interactions to enforce network access policies. The enforcement of such restrictions has been considered through SDN techniques in recent years by the research community. Furthermore, the enforcement approach has to deal with the *resource constraints* of IoT devices, which usually do not have enough computational power to use heavy cryptographic algorithms and security mechanisms. In addition, the enforcement mechanism has to be *as automated as possible* to increase the *scalability* of the approach and the *usability*, reducing (or even removing) any human interaction. An additional security challenge of IoT scenarios is how to ensure *auditability, data provenance, and verifiability* of the exchanged data while ensuring confidentiality in transactions.

To cope with these challenges, our approach leverages on different technologies for a comprehensive enforcement approach of the different security policies proposed through the extension of the MUD model. In particular, for the enforcement of network policies, we use SDN technology, which is strongly considered in current literature, as shown in Section 2, and it provides a high degree of automation. Furthermore, we use the CP-ABE cryptographic scheme to enforce data privacy, which provides the flexibility to protect data based on the combination of attributes. Furthermore, to deal with the expensive operations of CP-ABE, we propose the usage of a proxy (as described by Perez et al. [40]), so that IoT devices can delegate CP-ABE operations. For authorization policies over resources, we use a lightweight token approach combining CWT and AIF, which leverages the compact format of CBOR representation. Finally, to cope with trust and accountability challenges, our proposed framework has been integrated with blockchain technology, whereby encrypted IoT shared data are audited in a distributed way in the ledger, strengthening accountability and trust in IoT.

## 4. Augmenting Behavioral Profiles through MUD Extensions

As discussed above, the MUD model provides a way to reduce the attack surface of a specific device by limiting the communications from/to it. In particular, the network behavior is expressed in the form of policies or ACLs based on the YANG standard. The MUD model is composed by two main containers: “mud” and “acls”. The first one specifies high level information about the MUD profile, such as the MUD URL in which the MUD file is located (field “mud-url”), when it expires (“cache-validity”), when it was created (“last-update”), and its version (“mud-version”), as well as information about the device, such as the model (“model-name”) and firmware/software revision (“firmware-rev”, “software-rev”). Finally, this container enumerates the ACLs restricting the communications from/to the device, “to-device-policy” and “from-device-policy”. Then, the second container describes the details of the ACLs. Although the definition of the ACLS is based on the YANG data model for network ACLs, it has been augmented by the MUD standard to define more expressive restrictions. For example, the terms “manufacturer” and “same-manufacturer” enable the definition of policies to allow or deny the interaction with devices from the same manufacturer. Other fields allow referencing network components (e.g., “controller” or “local-networks”) without the need to know the associated IP addresses.

While the MUD data model provides a standardized and flexible way to specify network policies, the provided semantics does not allow the specification of more fine-grained security aspects. To cope with this, we extend the MUD model to accommodate more fine-grain aspects and four types of security policies: extended network access control (filtering) policies, channel protection, data privacy, and authorization over resources.

To realize such extended model, we embed MSPL features in the scheme. MSPL is usually employed to define security policies at medium-level abstraction (e.g., with information of the endpoints and communications, such as IP addresses and protocols), in an agnostic way to the enforcement process. In this regard, Listing 1 shows the extension of the fields *from-device-policy* (Line 2) and *to-device-policy* (Line 9) in order to allow the specification of MPLs, in addition to ACLs. In fact, *mspls/mspl* refers to the new MSPL module we propose (Lines 6 and 13), which includes a list of MSPLs.

Listing 1 **from-device/to-device policy extension for MSPL model.**

rw from-device-policy
  |  rw acls
  |    |  rw access-list* [name]
  |    |    rw name -> /acl:acls/acl/name
  |  rw mspls
  |       rw mspl-list* [name]
  |         rw name -> /mspl:mspls/mspl/name
rw to-device-policy
     rw acls
       |  rw access-list* [name]
       |    rw name -> /acl:acls/acl/name
     rw mspls
          rw mspl-list* [name]
            rw name -> /mspl:mspls/mspl/name

Listing 2 shows the main elements of the MSPL policy scheme. An MSPL policy is composed of a name (Line 5) and configuration (Line 6) that describes a particular capability (Line 7); for example, an MSPL policy can describe the authentication of the device. Apart from the capability, the configuration is also composed of a list of configuration rules (Line 8) as well as the priority of this security policy configuration (Line 13). In turn, each configuration rule defines the condition (Line 11) that must be accomplished in order to trigger the action (Line 10) of the rule. The priority field can be useful to resolve policy conflicts, deciding which policy should be enforced. Depending on the capability, conditions and actions are different so they have been modeled as independent modules for each capability, this is, for each capability, specific actions and conditions can be defined as different modules in order to provide extensibility, re-usability, and flexibility.

Listing 2 **MSPL module extension.**

module: umu-mspl-list
    |    rw mspls
    |        rw mspl* [name]
    |            rw name    string
    |            rw configuration
    |                capability    string
    |                configuration-rules
    |                    rw configuration-rule* [name]
    |                        |   rw configuration-rule-action
    |                        |   rw configuration-rule-condition
    |                    rw name
    |                    rw priority
end_module

Listing 3 shows the MUD extension with MSPL filtering rules. Although the MUD model also allows defining access control policies to filter the network traffic, MSPL extension permits defining additional aspects such as the MAC and IP addresses, as well as ports or interfaces. Nevertheless, with independence of the method to describe these policies (the MSPL extension or the usual MUD ACL), they are translated to SDN rules in order to enforce them in the SDN switches.

Listing 3 **Filtering extension.**

module: umu-mspl-filtering-condition
augment /mspl:mspls/mspl:mspl/mspl:configuration/mspl:configuration-
rules/mspl:configuration-rule:mspl:configuration-rule-condition
    |   rw packet-filter-condition
    |   rw application-layer-condition?
    |   qos-condition?
    |
end_module
module: umu-mspl-packet-filter-condition
augment /mspl:mspls/mspl:mspl/mspl:configuration/mspl:configuration-
rules/mspl:configuration-rule/mspl:umu-mspl-filtering-condition/
mspl:packet-filter-condition
    |   rw source-mac?
    |   rw destination-mac?
    |   rw source-address?
    |   rw destination-address?
    |   rw source-port?
    |   rw destination-port?
    |   rw direction?
    |   rw interface?
    |   rw protocol-type?
end_module

Listing 4 shows an example in order to extend configuration rule action and condition for data privacy policies. The specific privacy action (Line 2) adds the type of action (Line 5, e.g., data privacy) and the specific privacy method (Line 6). In fact, since there can be multiple methods (e.g., identity-based and attribute-based based privacy), each method can be defined as a new module (Line 7). In this case, as we enforce the data privacy policies using CP-ABE, we provide an example for attribute-based privacy which allows to include a list of key-value attributes (Lines 10–13). The CP-ABE policy that combines these attributes with AND statements is used to encrypt the published data. This means that only the devices that have all the required attributes will be able to decrypt such data. It should be noted that CP-ABE policies could be represented by more complex combination of attributes that can be considered in future implementation at the expense of increasing complexity.

Listing 4 **Data privacy extension.**

module: mspl-mspl-privacy-action
augment /mspl:mspls/mspl:mspl/mspl:configuration/mspl:configuration-
rules/mspl:configuration-rule:mspl:configuration-rule-action
    |   rw privacy-action-type    string
    |   rw privacy-method
end_module
module: umu-mspl-abprivacy-method
augment /mspl:mspls/mspl:mspl/mspl:configuration/mspl:configuration-
rules/mspl:configuration-rule/mspl:mspl-mspl-abprivacy-action/
mspl-privacy-method
    |   rw attributes
    |       rw attribute?*
    |           |   rw key    string
    |           |   rw value  string
    |       rw attribute-chain?    string
end_module

Furthermore, Listing 5 shows the MUD extension for authorization over resources policies. Whereas data privacy focuses on the access to data provided by IoT device, these policies are intended to protect the access to devices’ resources (e.g., /tmp). Here, the condition (Line 7) is reused from the filtering MSPL scheme, the AuthorizationSubject and AuthorizationTarget indicate to whom the rule applies, and the AuthorizationAction reflects the result of the rule (PERMIT/DENY). These policies are translated to XACML policy language, indicating the resource, the condition and the action, and stored in a database. The enforcement of these policies is performed through the XACML policy evaluation itself that will generate CWT-AIF credentials according to PERMIT decisions. That is, when a device attempts to access to another device’s resource, it asks for a token, which will be granted or not based on the XACML policies stored in the database.

Listing 5 **Authorization extension.**

module: mspl-mspl-AuthorizationAction
augment /mspl:mspls/mspl:mspl/mspl:configuration/mspl:configuration-
rules/mspl:configuration-rule:mspl:configuration-rule-action
    |   rw AuthorizationActionType
end_module
module: umu-mspl-AuthorizationCondition
augment /mspl:mspls/mspl:mspl/mspl:configuration/mspl:configuration-
rules/mspl:configuration-rule:mspl:umu-mspl-filtering-condition
    |   rw AuthorizationSubject   string
    |   rw AuthorizationTarget    string
end_module

Finally, Listing 6 shows an example of a channel protection policy. Whereas the condition (Line 34) can be also reused from the filtering schema, the action (Line 2) contains the capability-dependent information. Specifically, this kind of action allows modeling the channel protection technology (e.g., DTLS) as well as technology action parameters (e.g., ciphersuite and version), authentication parameters (e.g., pre-shared key (PSK) and certificate), and security properties (e.g., integrity algorithm) (Lines 6–9). By extending these fields as different modules (Lines 12 and 20), it provides flexibility for defining different technologies with different features of channel protection configurations (e.g., DTLS or IPSec). In the DTLS case, technology action parameters (Line 12) can be extended in order to represent specific technology parameters such as local and remote endpoints (Lines 15 and 16) of the security channel or basic information related with the ciphersuite or the version of the TLS that must be used (Lines 18 and 19). Authentication parameters (Line 20) are also extended for providing specific authentication values like required pre-shared key (Line 23) or certificates information (e.g., identifier, files, or paths, Lines 28–30).

Listing 6 **Channel protection extension.**

module: umu-mspl-data-protection-action
augment /mspl:mspls/mspl:mspl/mspl:configuration/mspl:configuration-
rules/mspl:configuration-rule/mspl:configuration-rule-action
  |   +--rw technology   string
  |   +--rw technology-action-parameters
  |   |  +--rw technology-parameter*
  |   |  +--rw authentication-parameters
  |   |   +--rw authentication-parameter*
  |   +--rw technology-action-security-properties
  |     +--rw technology-action-security-property
end_module
module: umu-mspl-dtls-technology-parameter
augment /mspl:mspls/mspl:mspl/mspl:configuration/mspl:configuration-
rules/mspl:configuration-rule/mspl:technology-action-parameters/
mspl:technology-parameter
  |   +--rw local-endpoint string
  |   +--rw remote-endpoint  string
  |   +--rw tunel boolean
  |   +--rw cipher-suite string
  |   +--rw ssl-version string
end_module
module: umu-mspl-authentication-parameters
augment /mspl:mspls/mspl:mspl/mspl:configuration/mspl:configuration-
rules/mspl:configuration-rule/mspl:technology-action-parameters/
mspl:authentication-parameters
  |   +--rw psk-value? string
  |   +--rw psk-path?  string
  |   +--rw ca-id?  string
  |   +--rw ca-path?  string
  |   +--rw ca-file?  string
  |   +--rw cert-id?  string
  |   +--rw cert-file?  string
  |   +--rw cert-path?  string
  |   +--rw pub-key-path?  string
  |   +--rw pub-key-file?  string
  |   +--rw pub-key-pass?  string
end_module
module: umu-mspl-data-protection-condition
augment /mspl:mspls/mspl:mspl/mspl:configuration/mspl:configuration-
rules/mspl:configuration-rule:mspl:umu-mspl-filtering-condition
end_module

It should be noted that, to define channel protection policies, it is necessary to specify additional aspects such as the used algorithms or the key length, which conform the confidentiality and integrity level. These aspects have been modeled independently so we are able to model the fields required according to the channel protection policy needs. Listing 7 shows examples for confidentiality and integrity security aspects. The module for confidentiality (Line 2) includes fields such as encryption algorithm (Line 5), key-size (Line 6), and operation mode (e.g., CCM, Line 7), whereas the integrity module (Line 8) allows specifying integrity algorithm (e.g., SHA, Line 11) as well as the type of integrity in terms of header or payload (Lines 12 and 13).

Listing 7 **Channel protection properties extension.**

module: umu-mspl-confidentiality-technology-action-security-property
augment /mspl:mspls/mspl:mspl/mspl:configuration/mspl:configuration-
rules/mspl:configuration-rule/mspl:technology-action-security-
properties/mspl:technology-action-security-property
  |   +--rw encryption-algorithm? string
  |   +--rw key-size?  string
  |   +--rw mode?  string
end_module
module: umu-mspl-integrity-technology-action-security-property
augment /mspl:mspls/mspl:mspl/mspl:configuration/mspl:configuration-
rules/mspl:configuration-rule/mspl:technology-action-security-
properties/mspl:technology-action-security-property
  |   +--rw integrity-algorithm? string
  |   +--rw integrity-header?  boolean
  |   +--rw integrity-payload?  boolean
end_module

Once we have defined the extension of the MUD standard model, the next section describes the main components and technologies we propose to obtain and enforce the policies of the extended MUD.

## 5. Architecture

**Figure 1:**
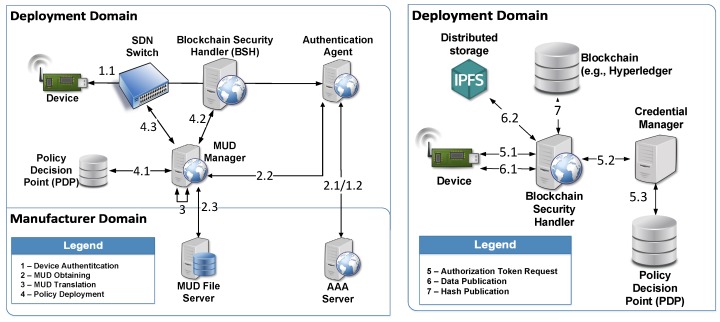
Proposed architecture.

The proposed architecture is described in Figure 1, in which different components are identified for obtaining and enforcing the extended MUD profiles. It should be noted that we consider two different domains, the *manufacturer domain*, in which the device and its MUD file are created, and the *deployment domain*, in which the device is installed. For the sake of simplicity, we divide the architecture into two figures, in which we additionally show the main processes of our approach. On the left, we show the architecture of the bootstrapping phase by which the MUD file is obtained by the deployment domain’s components. We understand the bootstrapping as “the phase of a smart object’s lifecycle in which it is installed and commissioned within a network” [41]. As part of this phase, we consider the device authentication, the obtaining and translation of the MUD file, and the deployment of the policies into different components to enforce the MUD restrictions. On the right, we show the architecture components required for enabling a secure data sharing process based on the extended MUD profiles, after the device has completed the bootstrapping process.

Our approach extends the architecture proposed by Matheu et al. [10], in which the MUD architecture was integrated in the bootstrapping phase. In this phase, we obtain the device’s information required to protect the device before it joins the network. Furthermore, it should be noted that we integrate the architectural components defined in the MUD standard, such as the *MUD Manager* (in charge of obtaining, translating, and enforcing the MUD file based on the MUD URL provided by the device), and the *MUD File Server*, which hosts the MUD files from a particular manufacturer.

As mentioned above, Figure 1 shows an overview of the main processes involved in our proposal that will be further detailed in Section 6. For the *Device Authentication* (Step 1) and *MUD Obtaining* (Step 2) phases, we use a similar approach to the one proposed by Matheu et al. [10], which is based on a combination of the Extensible Authentication Protocol (EAP) [42] and the Authentication, Authorization and Accounting (AAA) Framework [43]. Instead of using the Protocol for Carrying Authentication for Network Access (PANA) [44], we employ the Constrained Application Protocol (CoAP) standard as an EAP lower layer specifically designed for devices with constrained memory and computational resources [15]. Furthermore, the Remote Authentication Dial In User Service (RADIUS) [45] is used for the communication between the EAP authenticator and the EAP server, and it is employed to send the MUD URL after the device is authenticated.

Moreover, as in our previous work [10], we use an SDN approach based on the architecture defined in the EU H2020 project ANASTACIA [9] to enforce network access control restrictions included in the MUD file. In particular, we integrate some components of the SDN architecture into the MUD Manager, which represents the main building block of the MUD standard. We extend the MUD Manager functionality to support the *MUD Translation* (Step 3), and enforcement of flow rules into the network components (e.g., SDN switches). For this purpose, we use the OpenFlow protocol [7], so a SDN controller can install, modify, and remove SDN flows in such SDN switches. However, as described in Section 4, we extend the MUD data model to represent other security restrictions, beyond network access control, requiring additional entities to deploy and manage such policies. Therefore, after translating the MUD file, the set of restrictions is installed in different components during the *Policy Deployment* phase (Step 4). In particular, XACML and CP-ABE are used to translate authorization and data privacy policies, which are deployed in the *Policy Decision Point (PDP)* and *Blockchain Security Handler (BSH)*, respectively. After the device completes a successful bootstrapping process, and the different policies are deployed, it can perform its intended functionality by publishing their data (i.e., we consider a sensor device). For this purpose, an authorization token is required. Such credential can be obtained by the device through the *Authorization Token Request* process (Step 5). This process requires the evaluation of the XACML authorization policies before generating the access credential based on the CWT-AIF tokens.

For data publication, we provide data provenance and secure storage of the data generated by the devices through a combination of *blockchain (e.g., Hyperledger)* and scalable and distributed storage system, such as *IPFS*.

Blockchain not only offers a decentralized solution in which the immutability of the data is guaranteed, but also allows to trace in a public way the data generated by the devices, and mechanisms to verify the integrity and the provenance of such data. Blockchains are a subset of DLTs that represent the data as a chain of linked blocks. In our solution, we adopt permissioned blockchain as it facilitates the access control in the distributed ledger. Nonetheless, other DLT technologies (not necessarily blockchains, e.g., those DLT based on directed acyclic graphs instead of chains), might also be integrated in our architecture, as all of them are distributed and support auditability and provenance of shared data.

Our solution is intended to rule security and provenance in deployment domains that might belong to a Federated/Consortium of potentially large set organizations, and therefore, require permissioned blockchains that are not open to the general public. The consortium shares a distributed blockchain with multiple nodes that reach consensus on the content stored, assuming that there could be malicious or curious nodes. In particular, Hyperledger Fabric stands out for being a permissioned blockchain with a business approach, not attached to any cryptocurrency, which avoids speculation and miner fees. In addition, Hyperledger allows the creation of smart contracts of high functionality, and support Byzantine Fault Tolerance consensus mechanism to reach consensus between nodes with some potential number of malicious actors.

As mentioned above, the access to the blockchain is done through our trusted Blockchain Security handler component of the architecture, which is deployed in each deployment domain with proper rights to publish transactions in the permissioned blockchain. The IPFS network is used for saving the published data outside the blockchain network (i.e., *off-chain data*) to improve the scalability of blockchain network, and minimize security and privacy risks of publishing data in blockchain (even if encrypted). This way, the IPFS stores the real data during the *Data Publication phase* (Step 6), whereas the blockchain registers the data transaction of the IoT devices, saving *metadata* and a IPFS content identifier of published data (hash) during the *Hash Publication* stage (Step 7). Combining the Blockchain and the IPFS approach, we have two decentralized solutions working together to provide traceability and immutability of published data.

Based on the described technologies and protocols, the architecture comprises several entities and roles:

- *Device* represents the entity joining the network through a bootstrapping process. It acts as EAP peer and establishes an EAP session with the EAP Server, with the EAP authenticator as intermediary.
- *SDN Switch* is the entry point of the network. It is in charge of filtering the device traffic based on the network access control policies.
- *Authentication Agent*, which acts as EAP Authenticator, is an intermediate entity in the EAP communication between the device and the EAP Server.
- *AAA Server* acts as EAP Server to establish an EAP session with the device.
- *MUD Manager* is the main entity to manage MUD files, including the processes required for obtaining and enforcing the different security restrictions. It comprises several roles:
  - *IoT Controller* is intended to obtain the extended MUD file from the MUD File Server.
  - *Policy Interpreter* translates the extended MUD file to MSPL policies and specific configuration.
  - *SDN Controller* is in charge of enforcing the network access control policies of the extended MUD file in the SDN switch.
  - *Security Orchestrator* coordinates the enforcement of the extended MUD policies.
- *MUD File Server* is located in the manufacturer domain and stores the MUD files of the different devices.
- *Policy decision Point* is in charge of evaluating the authorization policies after they are translated from the MUD file.
- *Blockchain Security Handler* is in charge of managing the access over the blockchain in a transparent way for the devices. It comprises two roles:
  - *Proxy module* is in charge of enabling the access to the blockchain, and encrypting and publishing the data generated by the devices in the blockchain. It is also responsible for validating the CWT-AIF tokens to allow or not the access to the blockchain, as well as to enforce the data privacy and channel protection policies from the extended MUD.
  - *Authentication Module* is in charge of managing the token requests device, forwarding them to the Credential Manager.
- *Credential Manager* is responsible for managing a CWT-AIF token request and generating the token if the device is allowed, based on the policy evaluation in the PDP.
- *Distributed storage (IPFS node)* is in charge of storing the published encrypted data.
- *Blockchain (Hyperledger Fabric)* stores the transactions of the published data.

The interaction between the components of the different phases depicted in Figure 1 are further described in Section 6.

## 6. Message Exchange

As mentioned above, this section details the message exchange flow among the different components of the architecture previously discussed. According to the two pictures of Figure 1, we distinguish between the *bootstrapping* (picture on the left) and the *post-bootstrapping* phase (picture on the right) to describe the main processes.

### 6.1. Bootstrapping Phase

**Figure 2:**
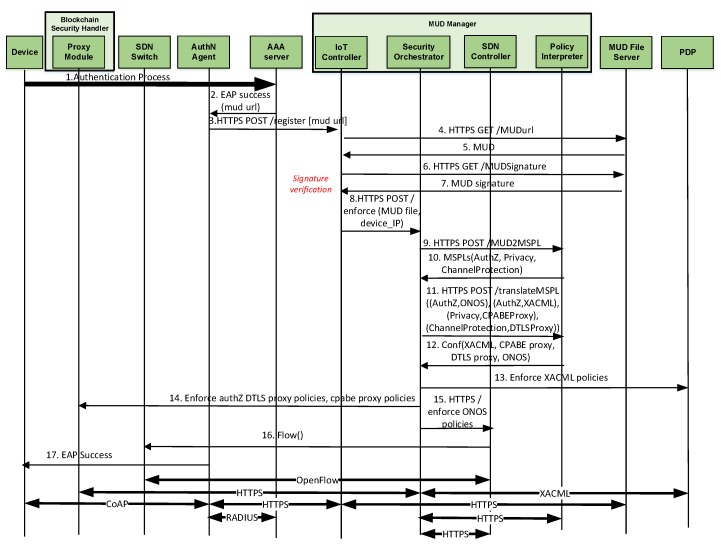
Message exchange of the bootstrapping phase.

The bootstrapping phase comprises the processes required to authenticate and protect the device and the network before accessing. As mentioned above, such processes include *Device Authentication*, *MUD Obtaining*, *MUD Translation*, and *Policy Deployment*. Figure 2 shows the messages exchanged in these processes, which are further described below.

#### 6.1.1. Device Authentication and MUD Obtaining

The device acting as CoAP client starts the bootstrapping by sending a CoAP POST /boot request to the CoAP Server, using CoAP-EAP. After an initial handshake, the exchange starts by using EAP-PSK [46] as authentication method. Further details of this exchange were provided by García-Carillo [15]. This exchange is grouped in Message 1 (*Authentication Process* of Figure 2). At the end of the exchange, the Authentication Agent receives cryptographic material from the AAA server along with the EAP Success message in an Access-Accept message. Although the MUD standard proposes mechanisms to communicate the MUD URL from the device, it leaves open the possibility for considering other options for constrained devices that cannot communicate the MUD URL, or in scenarios with limited Internet connection. As described by Matheu et al. [10], our approach proposes the usage of the AAA architecture to deliver the MUD URL. In particular, the MUD URL is embedded in the attribute “vendor-specific” of the RADIUS protocol (EAP Success, Message 2). The Authentication Agent does not forward immediately the EAP success to the device. Instead, it forwards the information of the MUD URL to the IoT Controller (as part of the MUD Manager) (Message 3). Then, this entity uses the MUD URL to contact the MUD File Server in order to get the MUD file (and an associated signature file) for the joining device (Messages 4–7). Furthermore, the IoT Controller verifies the MUD signature and requests the enforcement of the extended MUD file to the Security Orchestrator by sending such file (Message 8). It should be noted that a similar process was proposed in our previous paper [10] based on the MUD standard specification.

#### 6.1.2. Extended MUD Translation

After receiving the previous message, the Security Orchestrator requests the translation of the extended MUD file to a domain-specific extended MUD translator, that is, the Policy Interpreter. The standard MUD file is only able to describe network access control policies, expressed by ACLs, limiting the expressiveness of other security policy types. Our proposal extends the MUD model in order to specify different security behaviors, not only ACLs. Specifically, our proposed extended MUD file is composed by additional policies, including authorization (the access of the device to the network and resources is limited in the Proxy Module), data privacy (device must ensure privacy at data level), and channel protection (the communication between the device and the endpoint must be protected).

The translation of the extended MUD file requires additional information from the domain to translate the high level terms of the MUD such as “same-manufacturer” or “my-controller”. According to Figure 2, this information is obtained during the translation request from the Security Orchestrator (Message 9). Here, the Policy Interpreter retrieves the technological information from the endpoints involved in the extended MUD and identifies the main capability of the extended MUD policy (e.g., forwarding, channel protection, privacy, etc.). Then, for each capability, the Policy Interpreter instantiates a capability-specific policy modeled in MSPL, which is the policy language we use in our deployment for internal management and orchestration purposes. As it has a medium-level abstraction, the MSPL policies are interoperable and agnostic of the enforcement process (e.g., a filtering MSPL policy could be enforced in a traditional firewall or in an SDN network). In this case, the extended MUD file is translated in four different MSPLs (MSPL forwarding, MSPL authorization, MSPL data privacy and MSPL channel protection). These MSPL policies are forwarded to the Security Orchestrator (Message 10), which decides the best enforcement point for each one of them. According to the current deployment, the Security Orchestrator decides that the authorization policies will be enforced by using the deployed XACML-based PDP. As the devices are supposed to be constrained, they are not able to apply the privacy level specified, thus the Security Orchestrator decides that data privacy policies will be enforced in the Proxy Module by using a CP-ABE based solution. To do so, the CP-ABE policy is stored in the proxy module. The CP-ABE policy contains the combination of the attributes using AND statements (e.g., if the MUD extension requires that the data should be only accessible to UMU professors, the CP-ABE policy will be “UMU and professor”). For channel protection, devices implement the required crypto-suite, thus the Security Orchestrator will enable also the Proxy Module as DTLS channel protection endpoint. Then, the Security Orchestrator requests policy translations specifying the selected security enabler for each one of them (Message 11). We consider a security enabler as a software component in charge of implementing the security function associated with a MSPL policy, i.e., it allows enforcing the security policy in the managed system through a specific technology (e.g., filtering policies could be enforced through Netconf protocol [47] or SDN through Openflow [7]).

#### 6.1.3. Policy Deployment

Once the Security Orchestrator receives the final configurations for each enforcement point (Message 12), it requests the configurations enforcement to each security enabler. XACML configuration is enforced in the PDP in order to configure the authorization policies for the specific device (Message 13). CP-ABE and DTLS configurations are enforced in the Proxy Module. On the one hand, CP-ABE configuration prepares the proxy to encrypt the data received from the device by using the CP-ABE scheme to provide data privacy. On the other hand, DTLS configuration prepares the proxy to receive DTLS requests according to the specific cipher-suite defined in the security policy (Message 14). ONOS configuration is enforced by using ONOS northbound API which communicates with the SDN switches in order to provide the required connectivity (Messages 15 and 16). Once the policies have been enforced, the Security Orchestrator notifies the IoT Controller, which, in turn, notifies the Authentication Agent, which answers the EAP success to the device to complete the bootstrapping phase (Message 17). Table 1 summarizes the policies of the extended MUD, as well as the techniques and mechanisms for the translation and enforcement processes.

### 6.2. Post-Bootstrapping Phase

**Figure 3:**
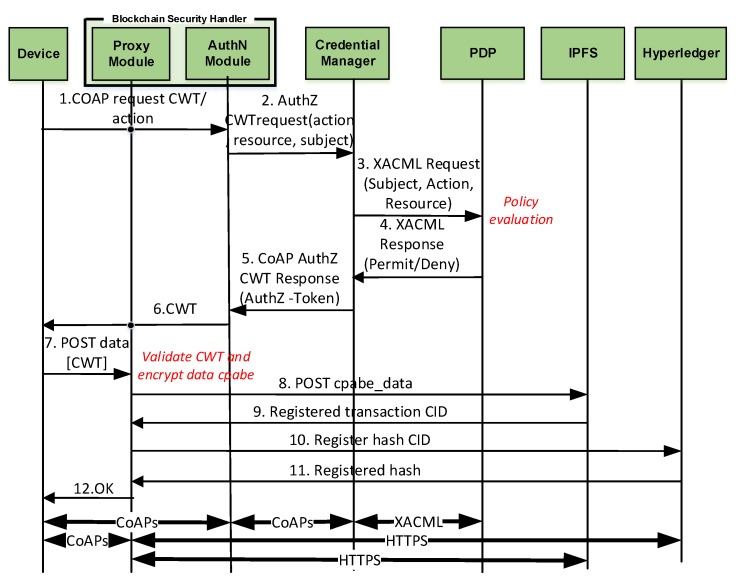
Message exchange of post-bootstrapping phase.

Once the device has been successfully bootstrapped, it starts to perform its intended operation by publishing their data. As already mention, we consider three main processes as part of this phase: *Authorization Token Request*, *Data Publication*, and *Hash Publication*. Figure 3 shows the detailed message exchange of these three processes. It should be noted that all device’s communications pass through the SDN switch. The next subsections detail step by step such processes.

#### 6.2.1. Authorization Token Request

To enforce the authorization policies over the resources (e.g., the access to the blockchain), we use an integrated approach based on XACML and authorizations tokens. As mentioned above, since the blockchain will be used by IoT devices with constrained capabilities, we use a Proxy Module as part of the Blockchain Security Handler that is in charge of providing the access of these devices to the blockchain. Furthermore, to cope with the requirements of resource constraints of IoT devices and networks, we use a lightweight approach based on CWT [13], which represent CBOR-encoded tokens [27], and the AIF format [28] to represent the access privileges in the token itself. Table 2 shows the resulting structure of CWT-AIF tokens. Furthermore, the table shows the value type for each claim and the numerical key used in the CBOR codification.

It should be noted that the resulting authorization token follows a similar approach to our previous capability-based access control approach [48]. In particular, we add a new claim (aif) to CWT, in order to integrate the AIF format with the aim to specify the resources and methods granted to the token’s holder. The AIF format follows a capability list structure, whose elements are the combination of a subject (the resource) and a list of actions (the methods). These pairs are specified with a URI [49] and a subset of CoAP methods. To reduce the size of the structure, AIF requires that the CoAPs methods are translated to their numerical expressions minus one. Then, the numerical set is combined into a single number by taking each number to the power of two and computing the inclusive OR of the binary representations of all the numbers.

As the original CWT, the proposed approach comprises three blocks:

- *COSE Header* with COSE parameters following the specification in [50]. It describes the cryptographic operations applied to the token and optional properties. These parameters can be cryptographically protected or not.
- *Payload*, containing the claims:
  - *Issuer* (iss) identifies the entity that issued the token in a string or URI format.
  - *Subject* (sub) represents the entity that is the subject (holder) of the token in a string or URI format.
  - *Audience* (aud) indicates the recipients that the token is intended for, in a string or URI array. If the entity processing the token is not indicated in this field, the token should be rejected.
  - *Expiration Time* (exp) specifies the expiration time, after which the token will not be valid, in a Numeric Date format.
  - *Not before* (nbf) represents the time, before which the token is not valid, in a Numeric Date format.
  - *Issued At* (iat) specifies the time when the token was issued, in a Numeric Date format.
  - *CWT ID* (cti) provides a unique identifier for the token, in a string format.
  - *Authorization Information* (aif) specifies the resources and methods the token allows to, by using the AIF format
- *COSE Signature*, *COSE MAC* or *COSE Encrypt*, depending on the protection mechanism of the token [50].

Based on this, when the device wants to access a certain resource (e.g., to publish data in the blockchain), it will make a request to the Authentication Module (as part of the Blockchain Security Handler) to obtain an authorization token (Step 1 in Figure 3). The Authentication Module will forward the request to the Credential Manager (Step 2), indicating the resource, action, subject, and the device attributes that were stored in the BSH during the bootstrapping. Then, the Credential Manager makes a XACML request to the PDP (Step 3), which will create a XACML response, based on the policies stored in the PDP database (or a Policy Retrieval Point according to XACML notation) that were translated from the extended MUD. The evaluation result is sent to the Credential Manager (Step 4). In the case the device is allowed, the Credential Manager generates a CWT-AIF token and forwards it to the Authentication Module and the device (Steps 5 and 6).

#### 6.2.2. Data and Hash Publication

As mentioned above, the privacy of the stored data required by the extended MUD policies is assured through the usage of the CP-ABE approach. During the bootstrapping, the CP-ABE policy is generated based on the attributes described in the extended MUD file and stored in the Proxy Module for further use during the post-bootstrapping phase. The CP-ABE policy is used to encrypt data in a way only the devices whose CP-ABE key contains the required attributes to access the data, are able to decrypt it. We consider that the Proxy Module also has an internal database to store the CP-ABE configuration. As the CP-ABE cryptography is too heavy for very constrained devices, we propose that the device delegates on the Proxy Module the CP-ABE operations. This way, it provides a privacy-preserving, yet accountable, fine-grain and user-controlled data sharing solution for constrained IoT deployments.

Following the message exchange of Figure 3, after obtaining the CWT-AIF token, the device can publish its information in the blockchain by using such token. For this purpose, the device sends a request to the Proxy Module (Step 7), which validates the CWT-AIF token. If the token is valid and the device is able to access the resource, the Proxy Module will encrypt the data with the CP-ABE policy, which embeds the required attributes for data protection of the extended MUD file. Once the data are encrypted to protect its privacy, the Proxy Module will request the publication of the data to the IPFS using a HTTP REST interface (Message 8). The IPFS will answer the proxy module with a content identifier (CID) obtained as a hash of the published data (Step 9). Then, the Proxy Module requests the registration of the CID and the metadata to the Hyperledger (Step 10). This way, the CID allows referencing the published data in a simple, public, and lightweight way, as the CID is shorter than the data itself. Finally, the Proxy Module will answer the device with an identifier of the transaction (Step 11), and finally, the device is also notified (Step 12). Furthermore, it should be noted that, while not shown in the figure, the Proxy Module is endowed with the APIs required by other devices to query the provenance information in blockchain and the shared encrypted data in the IPFS, as well as methods to assist devices by decrypting the data whenever needed.

## 7. Performance Evaluation

This section presents the evaluation of our proposal. For this purpose, we implemented and deployed the architectural components described in Section 5 as well as the processes described in Section 6.

### 7.1. Testbed

Table 3 provides an overview of the hardware and software used for each component and role. It should be noted that, for the implementation design, roles belonging to the same component may be implemented in different machines/devices.

As shown in Table 3, we integrated different existing libraries with our own implementations. In the case of the device, we simulated its behavior by using the Cooja Network Simulator (https://anrg.usc.edu/contiki/index.php/Cooja_Simulator), based on Contiki OS. Cooja requires a border router to enable the communication between the simulation environment and the external entities. The border router was also simulated in Cooja. Furthermore, we used our own implementation for the REST API of the SDN Orchestrator and the Policy Interpreter. To enforce the MUD network access control policies, we used ONOS Controller (ONOS: https://onosproject.org/), widely used in productions systems. The Policy Interpreter implemented the translator from MSPL into SDN low-level configurations understandable by ONOS controller.

Moreover, the blockchain implementation was based on Hyperledger Fabric 1.2 and the IPFS was the official Go IPFS implementation available in https://github.com/ipfs/go-ipfs. In both entities, the REST library used to manage the requests of the blockchain handler is Jersey (JAX-RS). As the blockchain is not the focus of the paper, the implementation was just a proof of concept to complete the scenario. Therefore, we used a single node with the SOLO consensus algorithm. Indeed, the blockchain is intended to be managed by the organization in which the device is deployed. Finally, for the CWT, we used our own Java implementation of the Credential Manager, supported by the CBOR library for the token translation and the Californium library for the implementation of CoAP-DTLS. The CP-ABE library used is available in https://github.com/junwei-wang/cpabe/.

### 7.2. Evaluation of the Proposal

**Figure 4:**
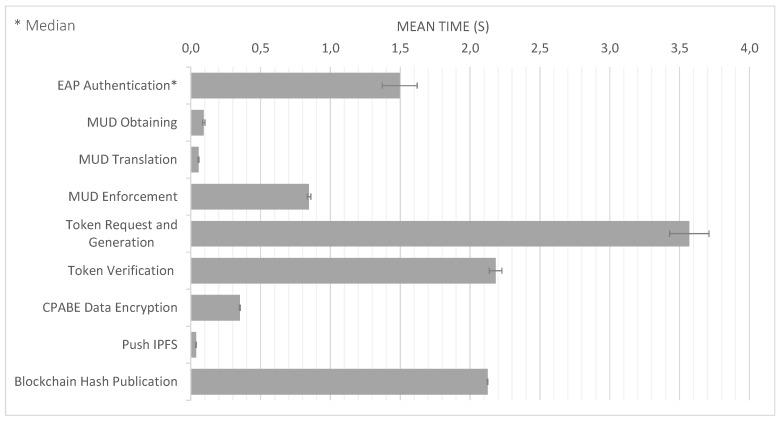
Time required for the different processes.

**Figure 5:**
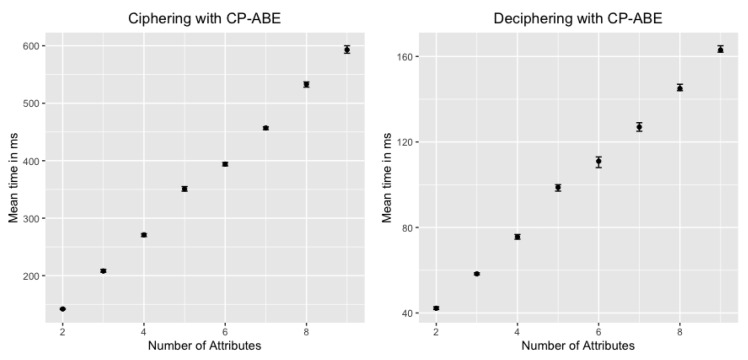
Encryption and Decryption time of CP-ABE.

Figure 4 compares the time required to complete the different phases of our approach:

- *EAP Authentication* comprises Messages 1 (set of messages CoAP-EAP), 2, and 17 in Figure 2. These messages require a median value of 1496 s. The reason to use the median is to provide a more accurate time, avoiding outliers due to packet retransmission. It should be noted that, for the other results, we employ the mean value. Although the EAP authentication is time consuming, this process is performed once, when the device is authenticated to join the network. In addition, the overload of the time with respect to the CoAP-EAP with EAP-PSK exchange [15] is minimum (30.5 ms). Indeed, as the MUD URL is transferred trough a non-constrained network, the extra time is negligible.
- *MUD Obtaining* evaluation was performed with a MUD file of 7.58 KB (see Appendix A), containing policies of the different types considered (network access control, authorization, channel protection, and data privacy). The delay for this stage is negligible (0.093 s) since the MUD file is also transferred trough a non-constrained network. This time includes the MUD obtaining as well as the MUD signature verification (Messages 3–7 of Figure 2).
- *MUD Translation* comprises Messages 8–12, including the MSPL translation, as well as the translation from MSPL to a specific configuration. In particular, the network access control policies are translated to ONOS configuration, the authorization policies to XACML policies, and the channel protection policies to DTLS configuration. Finally, data privacy policies are translated to a CP-ABE policy. This CP-ABE policy is obtained by combining the required extended MUD attributes for data privacy with AND statements. We use the same MUD file previously mentioned, thus it includes different types of policies. This MUD file (included in Appendix A) shows the data privacy policy (“umu-mspl-privacy-action:privacy-action-type”: “Data-Privacy”) named “MSPL0” with four attributes specified inside the field “umu-mspl-abprivacy-method:attribute” (“es”, “um”, “iot” and “dep5”). These data privacy attributes are combined with an AND statement to generate a CP-ABE policy to encrypt the published data. MUD translation time is also negligible with respect to other phases (0.055 s).
- *MUD Enforcement* time includes the delay required for deploying the different types of policies in the MUD file, including the DTLS and CP-ABE configuration in the proxy, the XACML authorization policies in the PDP, and the SDN enforcement of the network access control polices (Messages 13–16 in Figure 2). As the SDN enforcement involves the enforcement of the configuration with Openflow over the SDN switches, this enforcement is the most time-consuming step of this phase. Nevertheless, the time required is less than 1 s (0.847 s), which is affordable during the bootstrapping process.
- *Token request and generation* comprises Messages 1–6 of Figure 3. This phase includes the device request of the CWT-AIF token, the policy evaluation of the PDP, as well as the generation of the token in case of a PERMIT decision. The token was requested for the subject *bob*, the audience *coaps://CAFE:DCAF:8080*, and the access to the resource *tmp* with a POST operation. This operation is allowed by the PDP, as it was specified in the extended MUD (Appendix A). The token was generated with all the claims of the CWT in addition to the AIF extension, as well as the COSE header and the 256-bits signature with the ECDSA algorithm. As shown, this is the most time consuming phase (3570 s). The main reason for this is the involvement of the device for requesting the token through a constrained network. However, it should be noted that the token is meant to be reusable during its lifetime.
- *Token verification* involves Message 7 and the token verification process of Figure 3. The CWT used during the evaluation (in CBOR diagnostic notation) is shown in Listing 8. This CWT allows *bob* to access to the resource *tmp* through the POST method. This process requires a delay of 2182 s, which is strongly influenced by the message sent by the device, that is, the communication between the device and the Proxy Module. Indeed, the mean time consumed only by the verification process is 3044 ms.
- *CP-ABE data encryption* is referred as the encryption process in Figure 3, after Message 6. The CP-ABE implementation is based on the curve $y^{2}=x^{3}+3$ over the field $F_{p}$ for some prime p=3mod 4. The evaluation is performed by considering a 80-bit security level (i.e., |p|=512, |q|=160). For the results in Figure 4, we use asix-attribute CP-ABE policy as example. However, we further analyze the CP-ABE encryption time considering different number of attributes in Figure 5.
- *Push IPFS* comprises Messages 8 and 9 of Figure 3. The data published were temperature s measurements encrypted with CP-ABE, with the previous configuration, and it took 0.038 s.
- *Blockchain hash publication* comprises Messages 10–12 of Figure 3. The hash was obtained with the SHA256 algorithm. It is worth noting that the obtained time for this phase was measured in a test environment. For a complete evaluation of the hyperledger performance, the reader can consult the analyses performed by Hyperledger [51] and Jiang et al. [52].

Listing 8 **CWT+AIF token used in the evaluation performance.**

18([
    {1: −7},
    {1: “credentialmanager@odins.es”,
    2: “bob”,
    3: “coaps://CAFE:DCAF:5684”,
    4: 1579637954,
    5: 1579627954,
    6: 1579626954,
    7: h’64376B6B7036736E716D367430316D6E64763273676967633031’,
    8: [[“tmp”, 2]]}, h’4D4551434943786F32346B6F7A57496B4C7056464…’
]).

According to Figure 4, the publication over the blockchain is one of the most time-consuming phases. This is especially relevant because, depending on its purpose, the device could potentially publish its data very often. We reduced considerably this time by using the IPFS for storing the real data and the blockchain to store the hash linked to the real data. This way, the data published in the blockchain are shorter than the real data, reducing the publication time. Table 4 shows the time required to publish the data in the IPFS, and the delay for the data publication directly over the blockchain (without any type of encryption mechanism). By using the IFPS, the time reduction represents more than 340 ms (97.31%) compared to the publication over the blockchain.

Finally, Figure 5 shows a more detailed view of the encryption and decryption process of CP-ABE depending on the number of attributes used (2, 4, 6, 8, and 10 attributes). As shown, the time is proportional to the number of attributes used to encrypt or decrypt. It is worth noting that the number of attributes will usually be low (e.g., device manufacturer and model). It should also be noted that encryption is more expensive than decryption; for example, in the case of four attributes, the encryption time is 240 ms, whereas, for decryption time, it is 78 ms. For this reason, we delegate the encryption process in the Proxy Module. Some proposals consider more sophisticated approaches in which the encryption process is also delegated, such as the lightweight implementation of CP-ABE proposed in [40] (SymCPABE), which could be considered in an extension of our work.

**Figure 6:**
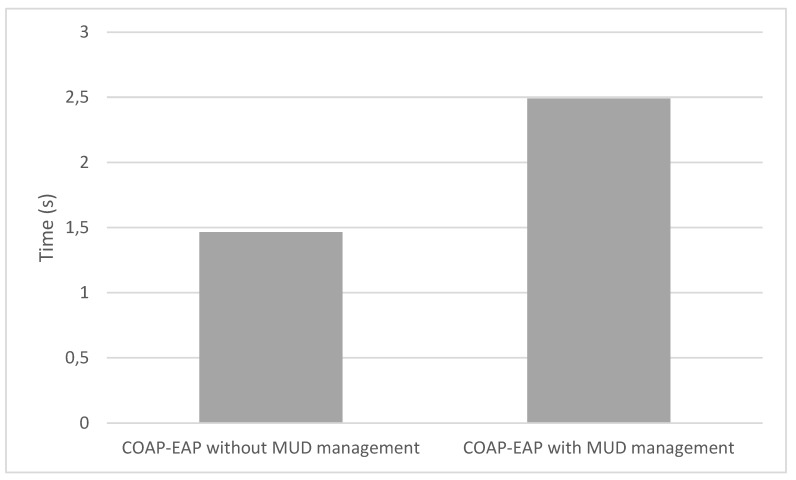
Overload of the MUD management during the bootstrapping.

**Figure 7:**
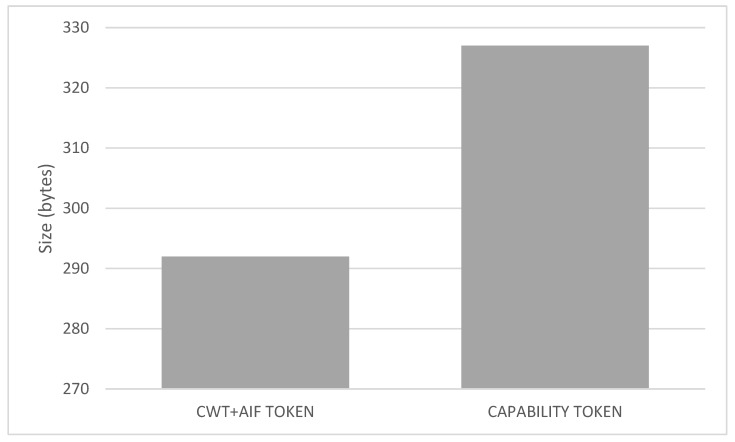
Overhead of our CWT-AIF token and the capability-based approach [48].

The overload of the processes, we add with respect to the bootstrapping and post-bootstrapping process are studied in Figure 6 and Figure 7. Figure 6 shows the comparison between a usual COAP-EAP bootstrapping process [15] for the device authentication, and the total time consumption with the additional processes to manage the MUD obtaining, translation, and policy deployment. As shown, the overload represents an extra delay of 1.0255 seconds with respect to the COAP-EAP bootstrapping process. Nevertheless, it should be noted that the bootstrapping process is performed only once, when the device joins the network, and, therefore, the overload does not affect to its normal operation. Moreover, Figure 7 shows the comparison between our token approach based on CWT and AIF, and the capability-based approach [48], which makes use of JSON representation. For the credential used in our tests (i.e., with the same access rights), our approach reduces in 35 bytes the JSON-based token (11.98% of the size). Due to the potentially huge number of IoT devices, and network restrictions, the resulting approach represents a lightweight and scalable solution for constrained scenarios.

## 8. Conclusions

The advent of increasing IoT-enabled scenarios poses new security challenges. In this context, proactive approaches are essential to reduce the attack surface and potential impact of a certain attack. This work describes an approach to enforce security restrictions during the bootstrapping process of an IoT device. For this purpose, we propose the usage of behavioral profiles by extending the current MUD standard to include not only network access control policies, but also data privacy, channel protection, and authorization policies. We also describe the processes required to obtain and enforce such behavioral profiles. Our approach integrates the use of SDN technologies with an access control infrastructure to ensure only legitimate and authorized entities get access to devices’ data and resources. Furthermore, we consider a blockchain platform to share the information provided by the devices through the use of Hyperledger. As future work, we plan to extend the proposed process in order to manage the whole security lifecycle of an IoT device, by integrating monitoring tools to react against potential attacks during IoT devices’ operation. 

## Figures and Tables

**Table 1:** Overview of the security policies.

Policy Type	Translation	Enforcement
Network access control	ONOS	SDNs
Channel protection	DTLS configuration	Proxy configuration
Data privacy	CP-ABE policies	Proxy configuration and CP-ABE encryption
Authorization over resources	XACML policies	XACML policy evaluation and CWT-AIF tokens

**Table 2:** Summary of the CWT+AIF claims.

Name	Key	Value Type
iss	1	text string
sub	2	text string
aud	3	text string
exp	4	integer or floating-point number
nbf	5	integer or floating-point number
iat	6	integer or floating-point number
cti	7	byte string
aif	8	AIF structure

**Table 3:** Testbed specifications.

Component	Hardware	Role	Software
Smart Object	Zolertia Z1 with 92 kB of nominal ROM and 8 kB of RAM	EAP peer	Cooja (Contiki OS 2.7)
		CoAP Client	cantcoap
		CoAP Server	cantcoap
Authentication Agent	Linux Ubuntu VM with 2 GB of RAM, 30 GB HDD and a processor Intel(R) Core(TM) i7-8550U at 1.9 GHz, using 1 core	EAP Authenticator	FreeRadius 2.0.2.
		CoAP Client	cantcoap
		CoAP Server	cantcoap
AAA Server	Linux Ubuntu VM with 2 GB of RAM, 30 GB HDD and a processor Intel(R) Core(TM) i7-8550U at 1.9 GHz, using 1 core	AAA Server	FreeRadius 2.0.2.
		EAP Server	C application
MUD Manager	Intel Core Processor (Haswell) at 1.5 GHz using 2vCores, 2 GB of RAM and 15 GB of HDD Intel(R) Core(TM) i7-2600 CPU at 3.4 GHz, using 3 vCores, 3.5 GB of RAM and 30 GB of HDD	IoT Controller	Python application
		Security Orchestrator	Django 2.2.2 and Falcon 2.0
		SDN Controller	ONOS
		Policy Interpreter	Django 2.2.2 and Falcon 2.0
MUD Server	Linux Ubuntu VM with 2 GB of RAM, 30 GB HDD and a processor Intel(R) Core(TM) i7-8550U at 1.9 GHz, using 1 core	MUD Server	Apache 2.4.39
Border router	Zolertia Z1 with 92 kB of nominal ROM and 8 kB of RAM	Border router	Contiki OS 2.7
Blockchain Security Handler	Windows 10 Pro with processor Intel(R) Core(TM) I7-7700K at 4.2 GHz, 24GB of RAM DDR4 and SSD M.2	Authentication Module	Jersey 1.19.1, Apache Tomcat
		Proxy Module	Jersey 1.19.1, Apache Tomcat, CP-ABE
PDP	Windows 10 Pro with processor Intel(R) Core(TM) I7-8550U at 1.8 GHz, 8GB of RAM DDR4 and 500 GB of SSD M.2	PDP	Apache Tomcat
Credential Manager	Windows 10 Pro with processor Intel(R) Core(TM) I7-8550U at 1.8 GHz, 8GB of RAM DDR4 and 500 GB of SSD M.2	Credential Manager	Java Application, cbor-2.4.1, Californium 2.1.0
Blockchain	Windows 10 Pro with processor Intel(R) Core(TM) I7-7700K at 4.2 GHz, 24GB of RAM DDR4 and SSD M.2	Blockchain	Hyperledger Fabric 1.2, Hyperledger Composer 0.20.7
IPFS	Windows 10 Pro with processor Intel(R) Core(TM) I7-7700K at 4.2 GHz, 24GB of RAM DDR4 and SSD M.2	IPFS	IPFS version 0.4.19

**Table 4:** Overload time between the IPFS and the blockchain (without CP-ABE encryption).

Publishing Mechanism	Mean Time (ms)	Confidence Interval
IPFS	9.5	4.52
Blockchain	352.75	30.24

## Appendix A. MUD File

Listing 9 MUD file used in the evaluation.

[{
    “ietf-mud:mud”: {
      “mud-version”: 1,
      “mud-url”: “https://iot_controller/iot_broker”,
      “last-update”: “2019-04-17T09:47:00+00:00”,
      “cache-validity”: 48,
      “is-supported”: true,
      “systeminfo”: “Wismote IoT device”,
      “mfg-name”: “Odins”,
      “documentation”: “http://doc.wismote.odins.com”,
      “model-name”: “iot_broker”,
      “from-device-policy”: {
        “access-lists”: {
          “access-list” [{
            “name”: “mud-55052-v6fr”
          }]
        },
        “mspl-list”:{
          “mspls”: [{
            “name”: “mud-55053-v6fr”
          },{
            “name”: “mud-55054-v6fr”
          }]
        }
      },
      “to-device-policy”: {
        “access-lists”: {
          “access-list”: [{
            “name”: “mud-55052-v6to”
          }]
        },
        “mspl-list”:{
          “mspls”: [{
            “name”: “mud-55054-v6to”
          }]
        }
      }
    },
    “ietf-access-control-list:acls”: {
      “acl”: [{
          “name”: “mud-55052-v6to”,
          “type”: “ipv6-acl-type”,
          “aces”: {
            “ace”: [{
              “name”: “ent0-todev”,
              “matches”: {
                “ietf-mud:mud”: {
                  “my-controller”: [null]
                },
                “ipv6”: {
                  “protocol”: 17
                },
                “udp”: {
                  “source-port”: {
                    “operator”: “eq”,
                    “port”: 5683
                  }
                }
              },
              “actions”: {
                “forwarding”: “accept”
              }
            }]
          }
        },
        {
          “name”: “mud-55052-v6fr”,
          “type”: “ipv6-acl-type”,
          “aces”: {
            “ace”: [{
              “name”: “ent0-frdev”,
              “matches”: {
                “ietf-mud:mud”: {
                  “same-manufacturer”: [null]
                },
                “ipv6”: {
                  “protocol”: 17
                },
                “udp”: {
                  “destination-port”: {
                    “operator”: “eq”,
                    “port”: 5683
                  }
                }
              },
              “actions”: {
                “forwarding”: “accept”
              }
            }]
          }
        }
      ]
    },
    “umu-mspl-list:mspls”: {
      “mspl”: [{
          “name”: “mud-55053-v6fr”,
          “type”: “ipv6-mspl-type”,
          “mspls”: {
            “mspl”: [{
              “name”: “MSPL0”,
              “configuration”: {
                “capability”: “Privacy”,
                “configuration-rules”:[{
                  “configuration-rule”: {
                    “configuration-rule-action”:{
                      “umu-mspl-privacy-action:privacy-action-type”:
                      “Data-Privacy”,
                      “umu-mspl-privacy-action:privacy-method”:{
                        “umu-mspl-abprivacy-method:attributes”:[
                          {”umu-mspl-abprivacy-method:attribute”:{
                            “umu-mspl-abprivacy-method:key”: “dc”,
                            “umu-mspl-abprivacy-method:value”: “es”}
                          },
                          {”umu-mspl-abprivacy-method:attribute”:{
                            “umu-mspl-abprivacy-method:key”: “dc”,
                            “umu-mspl-abprivacy-method:value”: “um”}
                          },
                          {“umu-mspl-abprivacy-method:attribute”:{
                            “umu-mspl-abprivacy-method:key”: “o”,
                            “umu-mspl-abprivacy-method:value”: “iot”}
                          },
                           {“umu-mspl-abprivacy-method:attribute”:{
                            “umu-mspl-abprivacy-method:key”: “ou”,
                            “umu-mspl-abprivacy-method:value”: “dep5”}
                          }
                        ]
                      }
                    },
                    “configuration-rule-condition”:{
                      “umu-mspl-filtering-condition:packet-filter-
                      condition”:{“umu-mspl-packet-filter-condition:
                      destination-address”:“my-controller”}
                    },
                    “name”: “Rule0”,
                    “priority”: 200
                  }
                }]
              }
            }]
          }
        },
        {
          “name”: “mud-55054-v6from”,
          “type”: “ipv6-mspl-type”,
          “mspls”: {
            “mspl”: [{
              “name”: “MSPL0”,
              “configuration”: {
                “capability”: “Protection_confidentiality”,
                “configuration-rules”:[{
                  “configuration-rule”: {
                    “configuration-rule-action”:{
                      “umu-mspl-data-protection-action:technology”:
                      “DTLS”,
                      “umu-mspl-data-protection-action:technology-
                      action-parameters”:[
                        {“umu-mspl-data-protection-action:technology-
                        parameter”:{“umu-mspl-dtls-technology-parameter:
                        remote-endpoint”: “my-controller”}
                      }],
                      “umu-mspl-data-protection-action:technology-action-
                      security-properties”:[
                        {
                          “umu-mspl-data-protection-action:technology-
                          action-security-property”:
                          {
                           “umu-mspl-confidentiality-technology-action-
                            security-property:encryption-algorithm”: “AES”,
                            “umu-mspl-confidentiality-technology-action-
                            security-property:key-size”: 128,
                            “umu-mspl-confidentiality-technology-action-
                            security-property:mode”: “CCM”
                         }
                        }
                      ]
                    },
                    “configuration-rule-condition”:{
                    },
                    “name”: “Rule0”,
                    “priority”: 200
                  }
                }]
              }
            }]
          }
        },
        {
          “name”: “mud-55054-v6to”,
          “type”: “ipv6-mspl-type”,
          “mspls”: {
            “mspl”: [{
              “name”: “MSPL0”,
              “configuration”: {
                “capability”: “Protection_confidentiality”,
                “configuration-rules”:[{
                  “configuration-rule”: {
                    “configuration-rule-action”:{
                      “umu-mspl-data-protection-action:technology”:
                      “DTLS”,
                      “umu-mspl-data-protection-action:technology-
                      action-parameters”:[
                        {
                        “umu-mspl-data-protection-action:technology-
                        parameter”:{
                            “umu-mspl-dtls-technology-parameter:
                            local-endpoint”: “my-controller”
                        }
                      }],
                      “umu-mspl-data-protection-action:technology-
                      action-security-properties”:[
                        {                         “umu-mspl-data-protection-action:technology-
                        action-security-property”:
                          {“umu-mspl-confidentiality-technology-action-
                          security-property:encryption-algorithm”: “AES”,
                           “umu-mspl-confidentiality-technology-action-
                          security-property:key-size”: 128,
                           “umu-mspl-confidentiality-technology-action-
                           security-property:mode”: “CCM”
                          }
                        }
                      ]
                    },
                    “configuration-rule-condition”:{
                    },
                    “name”: “Rule0”,
                    “priority”: 200
                  }
                }]
              }
            }]
          }
        }
{
          “name”: “mud-55055-v6fr”,
          “type”: “ipv6-mspl-type”,
          “mspls”: {
            “mspl”: [{
              “name”: “MSPL0”,
              “configuration”: {
                “capability”: “AuthoriseAccess_resurce”,
                “configuration-rules”:[{
                  “configuration-rule”: {
                    “configuration-rule-action”:{
                      “umu-mspl-authorization-action:AuthorizationActionType”: “ALLOW”,
                    },
                    “configuration-rule-condition”:{
                        “umu-mspl-filtering-condition:packet-filter-condition”:{
                        “umu-mspl-packet-filter-condition:source-address”:“IoT-device”,
                        “umu-mspl-packet-filter-condition:destination-address”:
                        “IoT-broker”,
                        “umu-mspl-packet-filter-condition:destination-port”: 5684
                      },
                      “umu-mspl-filtering-condition:aplication-layer-condition”:{
                       “umu-mspl-aplication-layer-condition:application-protocol”:
                       “CoAPs”,
                       “umu-mspl-aplication-layer-condition:method”:“POST”,
                       “umu-mspl-aplication-layer-condition:url”:“/tmp”
                      }
                    },
                    “name”: “Rule0”,
                    “priority”: 200
                  }
                }]
              }
            }]
          }
        }
      ]
    }
  }
]
